# Sorting and gene mutation verification of circulating tumor cells of lung cancer with epidermal growth factor receptor peptide lipid magnetic spheres

**DOI:** 10.1111/1759-7714.13625

**Published:** 2020-08-27

**Authors:** Sheng‐guang Wang, Bin Zhang, Chen‐guang Li, Jian‐quan Zhu, Bing‐sheng Sun, Chang‐li Wang

**Affiliations:** ^1^ Department of Lung Cancer Tianjin Medical University Cancer Institute and Hospital, National Clinical Research Center for Cancer, Key Laboratory of Cancer Prevention and Therapy, Tianjin’s Clinical Research Center for Cancer, Tianjin Lung Cancer Center Tianjin China

**Keywords:** Circulating tumor cells, *EGFR* gene mutation, EGFR peptide, lipid magnetic sphere, lung cancer

## Abstract

**Background:**

This study aimed to identify an efficient, simple, and specific method of detecting mutations in the epidermal growth factor receptor (*EGFR*) gene in isolated lung cancer circulating tumor cells (CTCs) and to improve the ability to obtain tumor tissue clinically.

**Methods:**

EGFR peptide lipid magnetic spheres (EG‐P‐LMB) were prepared by reverse evaporation, and characterization and cell capture efficiency assessed. The peripheral blood samples of 30 lung cancer patients were isolated and identified with the EG‐P‐LMB using 20 healthy volunteers as controls. Finally, the isolated CTCs were tested for *EGFR* gene mutations, and the tissue samples selected for comparison.

**Results:**

The prepared magnetic spheres had a smaller particle size and higher stability according to the particle size potential test. Their morphology was homogeneous by atomic force observation, and the UV test showed that there were peptides on the surface. The separation efficiency of EG‐P‐LMB was greater than 90% in PBS and greater than 80% in the blood simulation system. Compared with the tissue sample results, the positive rate of *EGFR* gene mutations was 94%. The CTC test results of 27 patients were consistent with the tissue test results of the corresponding patients, and the consistency with the tissue comparison test results was 90% (27/30).

**Conclusions:**

EG‐P‐LMB can effectively capture CTCs in the peripheral blood of patients with lung cancer. CTC detection can accurately identify mutations in the *EGFR* gene and improve the ability to obtain tumor tissue in clinical practice.

**Key points:**

**Significant findings of the study:**

EG‐P‐LMB can effectively capture CTCs in the peripheral blood of patients with lung cancer. CTC detection can accurately identify mutations in the *EGFR* gene and improve the ability to obtain tumor tissue in clinical practice.

**What this study adds:**

This study added EGFR peptide lipid magnetic spheres to capture CTCs in the blood. Genetic testing was performed and compared with tissues. It solves the problem of clinically difficult tumor tissue sampling.

## Graphical abstract

Significant findings of the study: EG‐P‐LMB can effectively capture CTCs in the peripheral blood of patients with lung cancer. CTC detection can accurately identify mutations in the *EGFR* gene and improve the ability to obtain tumor tissue in clinical practice. What this study adds: This study added EGFR peptide lipid magnetic spheres to capture CTC in the blood. Genetic testing was performed and compared with tissues. It solves the problem of clinically difficult tumor tissue sampling.

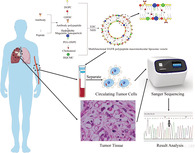

## Introduction

Lung cancer is the leading cause of cancer‐related death worldwide, and the non‐small cell lung cancer (NSCLC) subtype accounts for approximately 80% of all cases. The overall five‐year survival rate remains at approximately 15%, and most patients present with advanced disease.^1^ Early diagnosis and treatment has been reported to improve the five‐year survival rate by three‐ to four‐fold, with the possibility of curing the disease; however, more sensitive and specific methods are required.^2^, ^3^ In recent years, circulating tumor cells (CTCs) have become important potential biomarkers for the diagnosis, efficacy evaluation, and prognosis of several epithelial tumors, including lung cancer.^4^, ^5^ Due to the epithelial‐to‐interstitial transition, the recent CTC analysis method based on epithelial cell adhesion molecule (EpCAM) has a limited ability to detect CTCs in patients with malignant tumors.^6^

We have developed a self‐assembling peptide nanomicrocapsule with a lipid bilayer structure using EGFR‐binding peptide derivatives (an oligopeptide composed of 11 amino acids of GE11).^7^, ^8^, ^9^ The particle size and the content of the surface peptides can be controlled with different receptor targeting abilities in vitro and in vivo. Peptide ligands are a superior choice compared with antibodies or proteins given their advantages of low immunogenicity and easy preparation.^10^, ^11^, ^12^ The EGFR peptide may be a potential target for capturing CTCs in patients with NSCLC.^13^, ^14^ The purpose of this study was to explore the effectiveness and feasibility of CTC detection based on EGFR peptides in the diagnosis of NSCLC, particularly in early stages. This study provides technical reference for the early diagnosis, efficacy evaluation, prognosis, and micrometastasis detection in lung cancer patients. This minimally invasive biopsy technique has important clinical significance for the diagnosis and efficacy evaluation of lung cancer.

## Methods

### Sample source

Peripheral blood samples and tumor tissues of 30 patients with lung cancer were pathologically diagnosed in our hospital. The samples were collected from March 2018 to December 2019. Blood samples (7.5 mL) were collected from patients with medical anticoagulant blood vessels with the anticoagulant EDTA K2; these samples were stored and transported at normal temperature and tested within 72 hours. This study was approved by the ethics committee of our institution. The participants provided written consent after receiving verbal and written information regarding the study.

### Materials and instruments

Human NSCLC cell strains, including A549, NCI‐H1650, NCI‐H520, NCI‐H446, and NCI‐H1975, were obtained from the Shanghai Cell Bank of Chinese Academy of Sciences. The cells were cultured in RPM 1400 broth containing 10% newborn calf serum at 37°C and 5% CO_2_ in a constant temperature incubator. DMEM medium, fetal bovine serum, and trypsin were purchased from Gibco. EpCAM lipid magnetic spheres (Ep‐LMB) and EGFR‐LMB were purchased from JuKang (Shanghai) Bio‐Sci & Tech Co., Ltd. DSPE‐PEG was purchased from Avanti (USA). Fe_3_O_4_ particles, carboxymethyl chitosan cetyl quaternary ammonium salt (HQCMC), CK‐FITC, CD45‐PE, DAPI, and EGFR peptides were purchased from Huzhou Lieyuan Medical Laboratory Co., Ltd. A Prussian blue staining kit was purchased from Solarbio. 1,2‐dioleoylphosphatidylcholine (DOPC), dimethyl octadecyl epoxypropyl ammonium chloride (GHDC), cholesterol, dichloromethane, N‐hydroxysuccinimide (NHS), 1‐ethyl‐3‐(3‐dimethyl ammonium propyl) ammonium bicarbonate (EDC), and other commonly used reagents were purchased from Sinopharm (China). The BI‐90Plus laser particle size analyzer/Zeta potentiometer was purchased from Brooke‐Haiwen, USA. An XL‐30 environmental scanning electron microscope was purchased from PHILIPS, the Netherlands Company. A LDJ9600‐1 VSM magnetic performance tester was purchased from the American Digital Instrument Company. An OLYMPUS B × 61 fluorescence microscope was purchased from Olympus, Japan. The ultrasonic cell crusher model JY92‐IIDN and the rotary evaporator model XD‐52A were both purchased from Shanghai Bannuo Biotechnology Co., Ltd.

### Preparation of EG‐P‐LMB

EG‐P‐LMB was prepared according to the thin film method. PEG‐DSPE, cholesterol, DOPC, GHDC, HQCMC, and Fe_3_O_4_ particles were dissolved in dichloromethane with full dissolution first. After 0.1 mol/L PBS (pH = 7.4) was added, the mixed solution was ultrasonically emulsified using a probe type ultrasonic instrument with a power of 100 W (interval 1S for six minutes) at 25°C. After spinning and evaporating into a magnetic ball suspension, a 0.6 mg/mL EGFR peptide PBS solution was mixed with the addition of the coupling agents 1‐ethyl‐3‐(3‐dimethylaminopropyl) carbodiimide (EDC) and NHS. The mixture was stirred at 4°C for 24 hours at a constant rate. In the end, a lipid magnetic sphere modified with EGFR peptides was obtained and designated as EG‐P‐LMB.^13^, ^15^

### Characterization techniques

**Figure 1:**
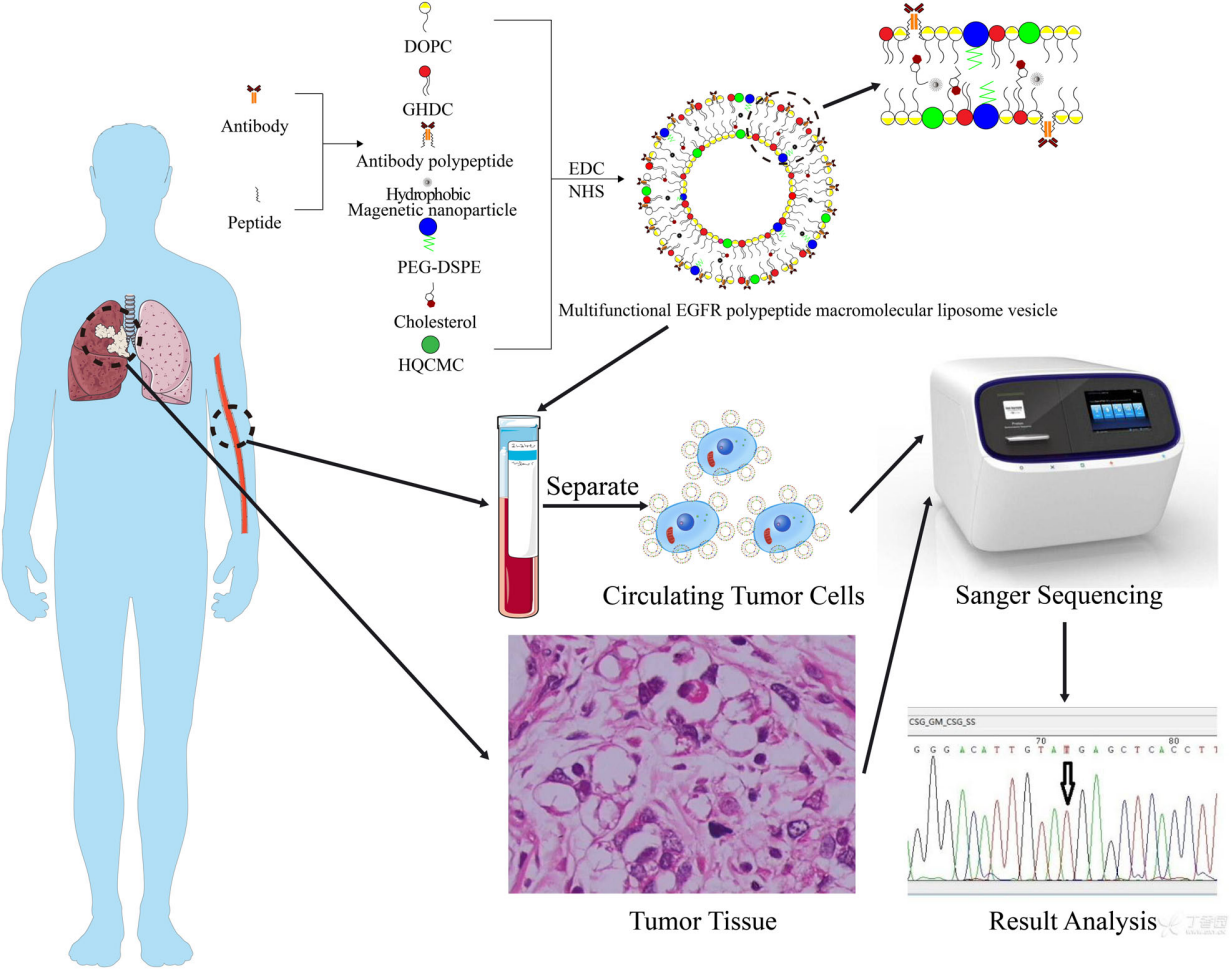
Flow diagram of EG‐P‐LMB preparation and detection.

The particle size of the lipid magnetic spheres was measured with a BI‐90Plus laser particle size analyzer/Zeta potentiometer. Then, 10 μL of the sample was diluted in 1 mL of distilled water and used for the particle size potential detection. The morphology of the lipid magnetic sphere was measured by atomic force microscopy (AFM). After taking 10 μL of the sample and diluting it in 1 mL of distilled water, 50 μL was applied to the mica plate and then measured after being dried. The ultraviolet absorption spectrum of the lipid magnetic sphere was detected by an ultraviolet spectrophotometer, and 10 μL of the sample was diluted in 1 mL of distilled water. The measurement was then performed immediately, and Ep‐IML and EG‐IML were detected in the same manner as EG‐P‐LMB (Fig 1).

### Cell capture efficiency

The lung cancer cell lines A549, NCI‐H1650, NCI‐H520, NCI‐H446, and NCI‐H1975 were routinely cultured in DMEM complete medium, and the complete medium contained 10% fetal bovine serum. The culture conditions were 37°C and 5% CO_2_ in a wet state. The following volumes of the culture solution were used: 2 mL of the culture solution was added to the 35 mm culture dish, 3 mL of the culture solution was added to the 60 mm culture dish, and 8 mL of the culture solution was added to the 10 cm culture dish.

A single‐cell suspension of the lung cancer cell line was prepared. After counting, 5, 10, 15, 20, and 25 μL lipid magnetic spheres were added to the A549 cells. The number of captured cells was counted, and the capture efficiency was calculated. Specifically, by checking the isolation and identification method of CTCs in the clinical samples below, the optimal volume of lipid magnetic spheres was added, and Prussian blue dye was added to observe the state of the cells captured by the magnetic spheres. Simultaneously, the capture efficiency was verified in other lung cancer cell lines, and A549 cells were added to the blood simulation system to verify the capture efficiency.

### Isolation and identification of CTCs in clinical blood samples of lung cancer

Whole blood (7.5 mL) of cancer patients was collected with an anticoagulative blood collection tube and centrifuged at 1500 rpm for 10 minutes (7.5 mL of peripheral blood from 30 lung cancer patients and 20 healthy volunteers). Following collection of the upper‐middle liquid into the 15 mL EP tube, PBS (pH = 7.4) was added and mixed well to reach a total volume of 7.5 mL. Then, 20 μL of EG‐P‐LMB was added and incubated at room temperature for 30 minutes, and was mixed once every five minutes. The centrifugal tube was inserted into the magnetic separator to absorb for 15 minutes, after which the supernatant was discarded and the EP tube was removed. PBS was used to perform a magnetic separation wash on the captured CTCs. Then, 30 μL of DAPI, 30 μL of CK19‐FITC, and 10 μL of CD45‐PE were added and mixed uniformly and stained for 15 minutes without light. After staining, 1 mL of ddH_2_O was added, magnetic separation was performed for 15 minutes on the magnetic separation plate, and the supernatant was discarded. Finally, 30 μL of ddH_2_O was added to the EP tube for suspending, smeared evenly on the center of the 3‐Aminopropyitriethoxyssicane(APES) glass slide when mixed, and observed by fluorescence microscopy.

### Gene mutation detection

The CTCs and tissue samples captured in all patients were genetically tested. The *EGFR* genetic testing was performed by the Huzhou Lieyuan Medical Laboratory Co., Ltd. Genomic DNA was extracted from the tumor samples using the QIAmp DNA Minikit (QIAGEN, CA) according to the manufacturer's instruction, and the obtained DNA was stored at −80°C for a long period of time. Primers, applied Primer5, and 01i906 software were designed to evaluate the feasibility of the primers. The following primer sequences were synthesized by Sangon Biotech (Shanghai) Co., Ltd.: *EGFR* 19 forward: 5′‐GCCTAGACGCAGCATCATTA‐3′, reverse: 5′‐ATGCCTCCATTTCTTCATCC‐3′; *EGFR* 21 forward: 5′‐GTAAGTTCAAGCCCAGGTCT‐3′, reverse: 5′‐GCAAGTACTGTTCCCAAAGC‐3′. PCR was conducted with the following steps: 1.5 μL of DNA product, 0.75 μL of forward and reverse primers, 15 μL of mix, and ddH_2_O were added to reach a total volume of 30 μL. The PCR program used a predenaturation temperature of 94°C for three minutes; followed by denaturation at 94°C for 15 seconds, annealing at 60°C for 20 seconds, and primer extension at 72°C for one minute for a total of 35 cycles; the supplementary extension stage consisted of five minutes at 72°C. Gel electrophoresis was performed with a 2% agarose gel; the electrophoresis bands were observed using a full‐function luminescence and fluorescence bioimage analysis system. The *EGFR* gene mutations were detected by Sanger sequencing if a band was present. The sequencing results were compared in the NCBI‐BLAST database for the mutational analysis.

### Statistical analysis

All statistical analyses in this study were performed using SPSS 21.0 software. The differences were statistically significant at *P* < 0.05.

## Results

### Characterization test

**Figure 2:**
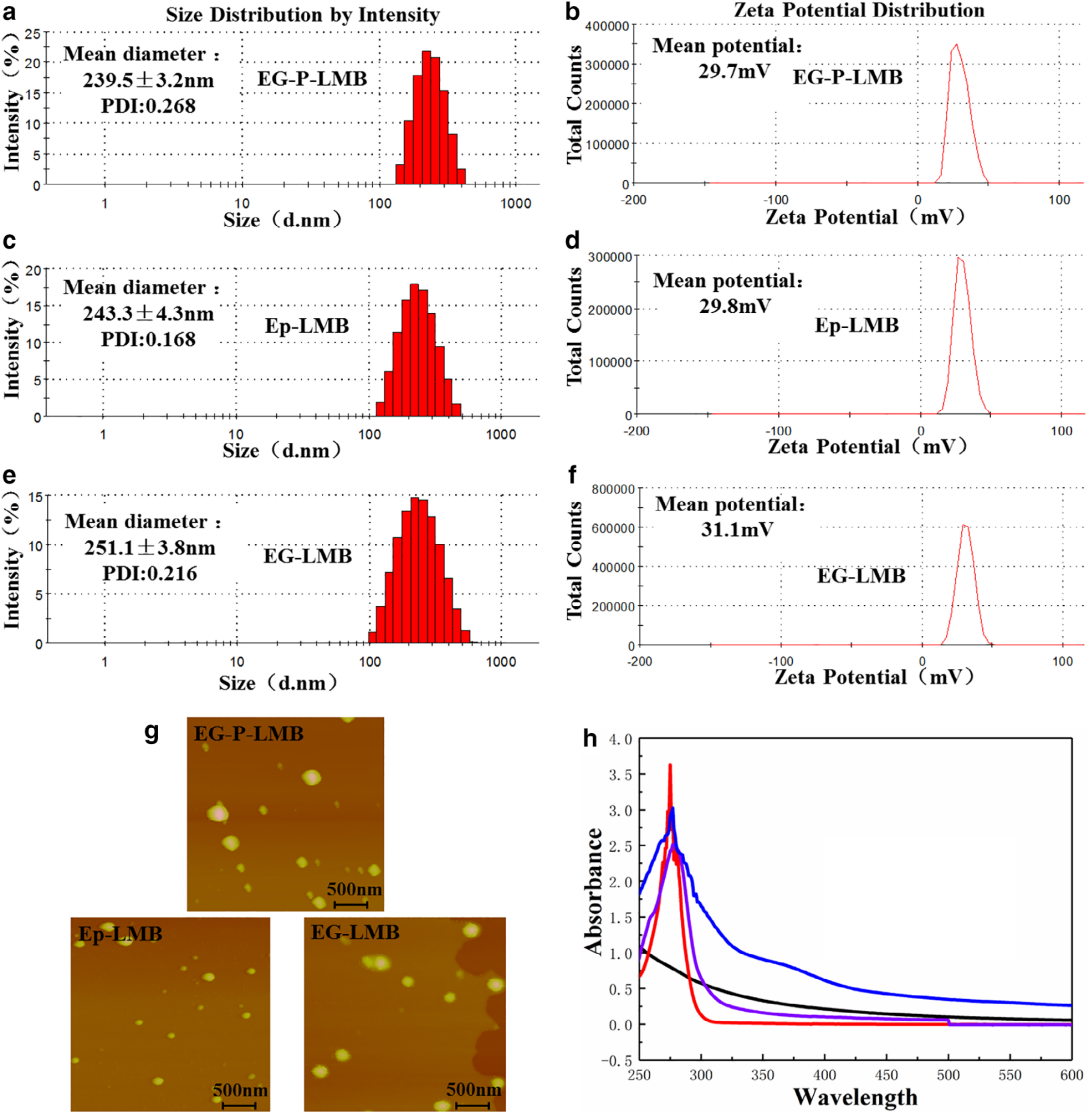
The characterization test results of EG‐P‐LMB, Ep‐LMB, and EG‐LMB. (**a**) EG‐P‐LMB particle size distribution diagram; (**b**) EG‐P‐LMB potential distribution diagram; (**c**) Ep‐LMB particle size distribution diagram; (**d**) Ep‐LMB potential distribution diagram; (**e**) EG‐LMB particle size distribution diagram; (**f**) EG‐LMB potential distribution diagram; (**g**) the results of the atomic force test of lipid magnetic spheres; and (**h**) ultraviolet test results of lipid magnetic spheres 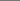, LMB; 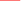, EG‐P‐LMB; 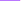, Ep‐LMB; 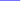, EG‐LMB.

The particle size detection and zeta potential analysis results of EG‐P‐LMB, Ep‐IML, and EG‐IML are shown in Fig 2. Fig 2a shows the particle size test chart of EG‐P‐LMB, which had an average particle size of 239.5 ± 3.2 nm. The measured solution had the smallest particle size. The size of the particles in the solution determines the stability of the solution, and the prepared lipid magnetic spheres solution had a particle size distribution between 141.8 and 396.1 nm and a polydispersity index (PDI) of 0.268. The diameter distribution was narrow, indicating that the particle distribution is evenly distributed compared with the particle size results of Ep‐LMB (Fig 2c) and EGFR antibody lipid magnetic spheres (EG‐LMB) (Fig 2e). Fig 2b shows the potential diagram of EG‐P‐LMB with a positive charge of +29.7 mV. The positively charged lipid magnetic spheres can be easily dispersed in the hydrophilic solution. Thus, the EG‐P‐LMB results were essentially consistent with the potential detection results of Ep‐LMB (Fig 2d) and EG‐LMB (Fig 2f). Fig 2g shows that the three types of immunomagnetic microspheres were spherical in shape and of different sizes; furthermore, there was no agglomeration, indicating that the immunomagnetic microspheres had good stability and a regular shape. The particle size measured by AFM was approximately 250 nm, which is consistent with the distribution range of particle size detection. Fig 2h shows the results of the UV test. LMB had no absorption peak at 280 nm whereas EG‐P‐LMB, Ep‐LMB, and EG‐LMB all had a wide absorption peak at 280 nm, which reflects the characteristics of the protein and indicates that EpCAM, EGFR antibodies, and the EGFR peptide were indeed attached to the surface of the magnetic sphere.

### Cell capture efficiency analysis

**Figure 3:**
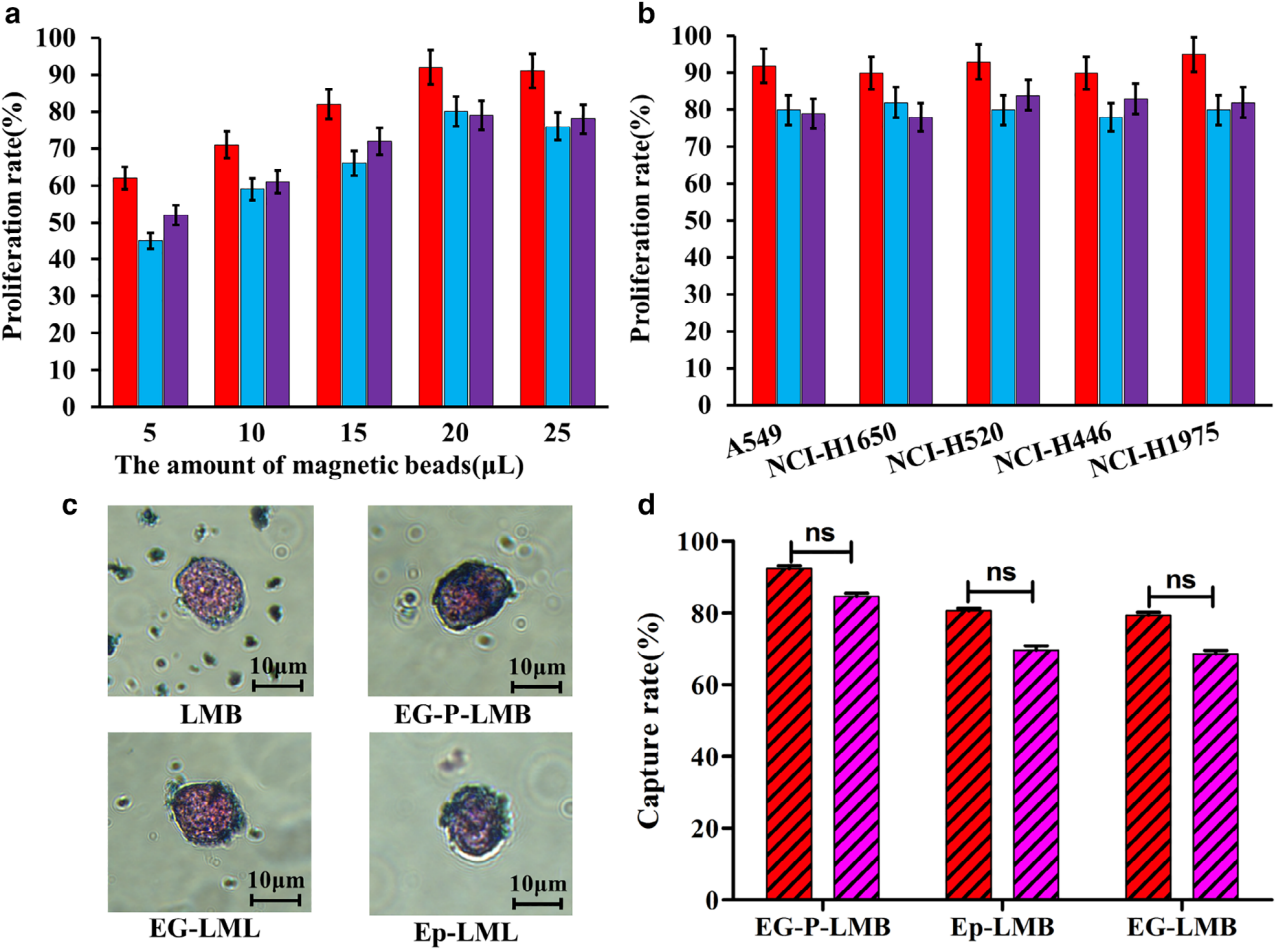
In vitro simulation of CTC capture efficiency. (**a**) The capture efficiency of EG‐P‐LMB, EP‐LMB, and EG‐LMB for A549 cells in the PBS system 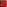, EG‐P‐LMB; 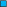, Ep‐LMB; 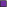, EG‐LMB; (**b**) capture efficiency of EG‐P‐LMB, EP‐LMB, and EG‐LMB at 20 μL for different cancer cell lines 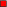, EG‐P‐LMB; 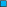, Ep‐LMB; 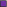, EG‐LMB; (**c**) distribution of the four types of magnetic beads on the cell surface; and (**d**) total cell capture rate of the three types of magnetic beads in the PBS and blood systems 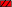, PBS; 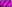, Blood.

The capture efficiency of adding different volumes of magnetic spheres to the PBS system is shown in Fig 3a. When adding different volumes of magnetic spheres, EG‐P‐LMB always displays the highest capture efficiency of the different magnetic spheres; moreover, the highest EG‐P‐LMB capture efficiency occurs when 20 μL of magnetic spheres are added. Fig 3b shows the capture efficiency of different magnetic spheres with different lung cancer cell lines. The results show that EG‐P‐LMB had the highest capture efficiency in each cell line, with an average capture efficiency of 92%; Ep‐LMB and EG‐LMB had average capture efficiencies of 80% and 81.2%, respectively. The Prussian blue staining results of the cells captured by magnetic spheres are shown in Fig 3c. As shown, LMB is not specific to lung cancer cells, which display many magnetic spheres distributed around the cells and only a small amount of LMB enriched on the cell surface. EG‐P‐LMB, Ep‐LMB, and EG‐LMB all displayed good specificity with many magnetic spheres enriched on the cell surface. Fig 3d shows the capture efficiency in both the PBS system and the simulated blood system. The results show that EG‐P‐LMB displayed the highest capture efficiency, with a rate over 90%, but the capture efficiency decreased in the simulated blood system due to the viscous blood and interference of more white blood cells. Thus, EG‐P‐LMB has a higher capture efficiency and can more effectively capture CTCs in the peripheral blood of patients with lung cancer.

### Immunofluorescence identification of CTCs in clinical blood samples of lung cancer patients

**Figure 4:**
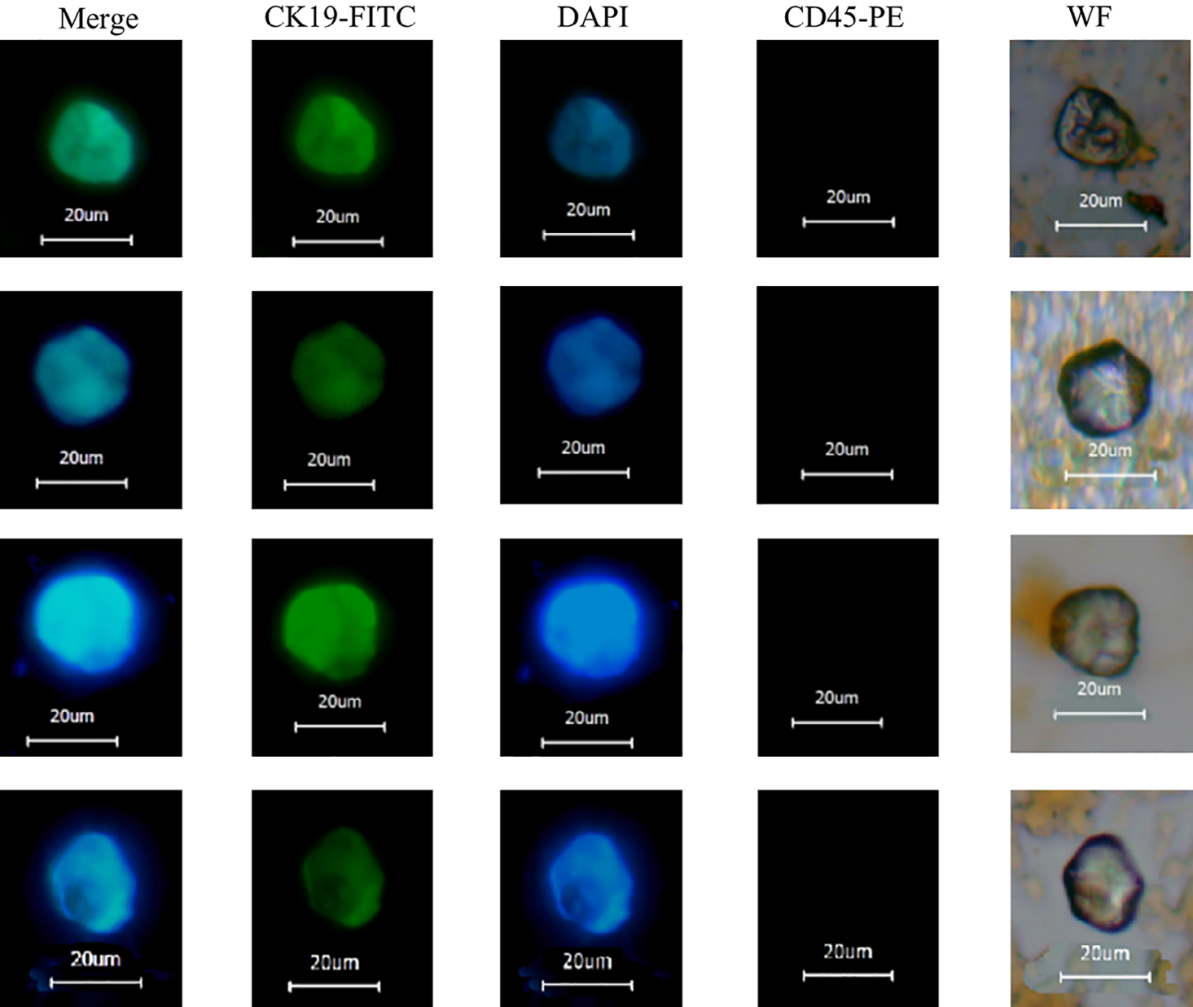
CTC immunofluorescence in the peripheral blood of patients with lung cancer.

The lung cancer CTCs isolated from EG‐P‐LMB were subjected to smear observation, as shown in Fig 4, in which there was obvious cell morphology under white light, CK19‐FITC green fluorescence staining was positive, DAPI blue fluorescence staining was positive, and CD45 cells with no fluorescence were considered CTCs.

### *EGFR* gene mutation test results

We constructed the magnetic sorting system of lung cancer CTCs by preparing EG‐P‐LMB. CTCs were captured from the peripheral blood of 30 patients with lung cancer using EG‐P‐LMB, and the captured CTCs were analyzed for *EGFR* gene mutation. When comparing the CTC test results with clinical features of the patients (Table 1), the *P*‐value was greater than 0.05; thus, the *EGFR* mutation was not related to age, gender, smoking history, clinical stage, metastasis, or histological subtype.

**Table 1:** Relationship between *EGFR* gene mutations and clinical features in patients with lung cancer

Clinical features			
	*EGFR* mutation	No *EGFR* mutation
Mutation	(*n* = 16)	(*n* = 14)	*P‐*value
Age			
Median	68	65	
Mean	65	64	
Range	41–86	36–87	
Gender			
Male	3 (19%)	4 (29%)	0.87
Female	13 (81%)	10 (71%)
Smoking			
Before and now	14 (88%)	6 (43%)	0.88
Never	2 (12%)	8 (57%)
Stage			
I	7 (44%)	3 (21%)	0.73
II	3 (19%)	3 (21%)	
III	4 (25%)	6 (43%)	
IV	2 (13%)	2 (14%)	
Tumor site			
No transfer	16 (100%)	13 (93%)	0.93
Metastasis	0 (0%)	1 (7%)
Hyphology			
Adenocarcinoma	6 (38%)	5 (36%)	0.84
BAC or adenocarcinoma with BAC feature	9 (56%)	8 (57%)	
Other	1 (6%)	1 (7%)	

Statistically significant at *P* < 0.05.

**Figure 5:**
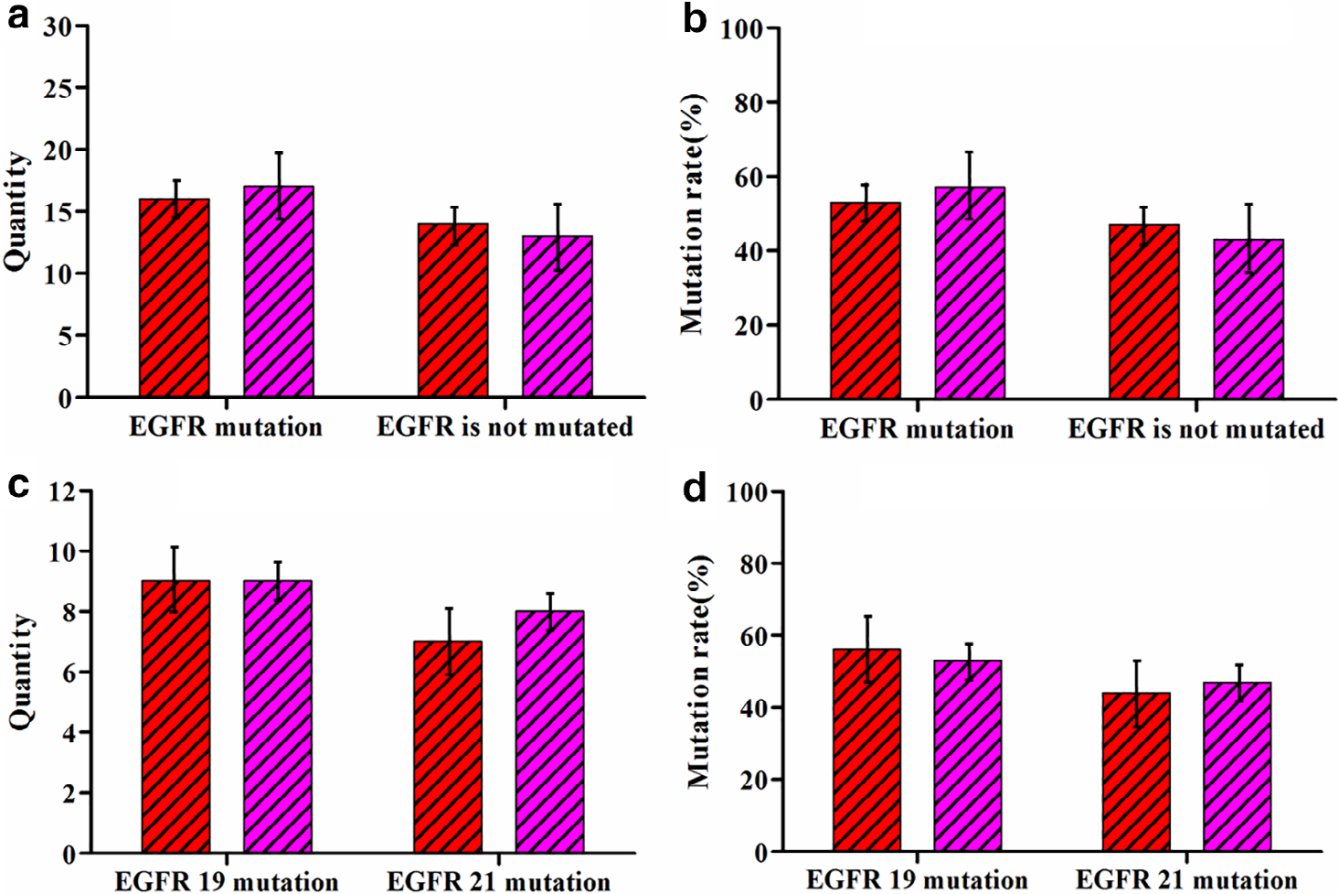
Analysis of *EGFR* gene mutation in CTCs and tissue samples from 30 patients. (**a**) Comparison of positive results of *EGFR* gene mutations in CTCs and tissue samples 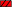, CTC; 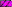, Organization; (**b**) comparison of the positive rate of *EGFR* gene mutations in CTCs and tissue samples 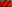, CTC; 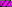, Organization; (**c**) comparison of the number of *EGFR* 19 and *EGFR* 21 mutations in CTCs and tissue samples 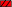, CTC; 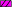, Organization; and (**d**) comparison of the incidence of *EGFR* 19 and *EGFR* 21 mutations in CTCs and tissue samples 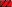, CTC; 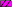, Organization.

The CTC samples isolated from the blood of 30 patients were detected by gene sequencing. All the tests were completed (Table 2 and Fig 5), and 16 cases tested positive for *EGFR* mutations. The positive detection rate of mutations was 53% (16/30). There were nine mutations in the *EGFR* 19 exon, corresponding to a mutation rate of 30% (9/30); these mutations were mainly located in del2239‐2253. Mutations in the *EGFR* 21 exon were mainly detected in L858R and occurred with a mutation rate of 23% (7/30). Positive *EGFR* mutations were detected in 17 of the patients’ tissue samples, corresponding to a detection rate of 57% (17/30). Among those, mutations in *EGFR* 19 were detected in nine samples, with a mutation rate of 30% (9/30), and mutations in *EGFR* 21 were detected in eight samples, with a mutation rate of 27% (8/30). Thus, of the samples with mutations, the mutation rate of *EGFR* 19 was 56% (9/16), the mutation rate of *EGFR* 21 was 44% (7/16), and the mutation rate of *EGFR* 19 was 53% (9/17), the mutation rate of *EGFR* 21 was 47% (8/17), and there was no statistical difference (*P* > 0.05). Based on the tissue detection results, the positive detection rate of *EGFR* gene mutations in CTCs was 94% (16/17). Additionally, the CTC detection results of 27 patients were consistent with the tissue detection results of the corresponding patients, with a consistency of 90% (27/30).

**Table 2:** CTC gene mutations and tissue mutations in lung cancer patients

*EGFR* mutation			
	CTCs	Tumor tissue	
Sample type	(*n* = 16)	(*n* = 17)	*P*‐value
*EGFR* 19 mutation	9 (56%)	9 (53%)	0.69
*EGFR* 21 mutation	7 (44%)^†^	8 (47%)^†^ ^‡^	

Statistically significant at *P* < 0.05,

^†^ Genetic detection results of CTCs and tissues from two samples were inconsistent.

^‡^ Mutations were detected only in tissue but not in CTCs of one sample.

## Discussion

The sensitive, specific detection and enumeration of CTCs remains a challenge in patients with NSCLC; however, this is still a developing field, with no universal method of detection suitable for all types of cancer.^16^, ^17^ Currently, a more mature technology commercially available for identifying CTCs from the blood of cancer patients is CellSearch, which uses immunomagnetic beads^18^, ^19^; other capture technologies, including array scanning, functionalized micro‐nanostructured surfaces, microfluidic micro‐mixers, etc are also being developed and applied.^20^ Among them, immunochemical‐based beads or nanoparticles can recognize and capture CTCs in whole blood with high efficiency and high selectivity. The CTC capturing method of the CellSearch system is based on EpCAM antibody‐coated magnetic nanoparticles. Considering the limitations of antibody‐based CTC capture technology, it is not possible to capture CTCs lacking the EpCAM protein or CTCs that have undergone the epithelial‐mesenchymal transition and other CTCs such as high EGFR.^21^, ^22^, ^23^, ^24^

In this study, EGFR peptides with a higher affinity were used to modify magnetic spheres to isolate EGFR phenotype CTCs and detect *EGFR* gene mutations in these isolated CTCs. A new CTC analysis method based on EGFR peptides was developed. We prepared EG‐P‐LMB and compared them with Ep‐LMB and EG‐LMB for specific targeting and rapid isolation of cancer cells. The preparation process of EG‐P‐LMB was also discussed, and the magnetic ball with the best capture efficiency was selected to perform the CTC capturing and identification of clinical lung cancer blood samples. Genetic testing was conducted to study its clinical applicability. The results indicated that EG‐P‐LMB was more effective in capturing CTCs in the peripheral blood of lung cancer patients. The CTCs can accurately detect *EGFR* gene mutations and can solve the problem of obtaining tumor tissue in clinical settings. The study provides technical reference for early diagnosis, efficacy evaluation, prognosis, and micrometastasis detection for lung cancer patients. This minimally invasive biopsy technique has important clinical significance for the diagnosis and efficacy evaluation of lung cancer.

In summary, the detection results of *EGFR* gene mutations in CTCs were essentially consistent with those in tissues. EG‐P‐LMB was more effective in capturing CTCs in the peripheral blood of lung cancer patients. CTCs can detect mutations in the *EGFR* gene accurately and could solve the problem of obtaining tumor tissue in clinical settings. Thus, CTC detection can be used as a new lung cancer cell identification method by providing a technical reference for early diagnosis, efficacy evaluation, prognosis, and micrometastasis detection for lung cancer patients. Meanwhile, as a minimally invasive biopsy technique, it also has huge clinical significance for the diagnosis and efficacy evaluation of lung cancer.

## Disclosure

The authors declare that there are no conflicts of interest.
